# Critical success factors for adopting green supply chain management and clean innovation technology in the small and medium-sized enterprises: A structural equation modeling approach

**DOI:** 10.3389/fpsyg.2022.1008982

**Published:** 2022-11-03

**Authors:** Shoaib Maqsood, Yan Zhou, Xintong Lin, Shuai Huang, Ihsan Jamil, Khurram Shahzad

**Affiliations:** ^1^School of Business, Qingdao University, Qingdao, China; ^2^School of Economics and Finance, Xi'an Jiaotong University, Xi'an, China

**Keywords:** green supply chain management (GSCM), clean innovation technology (CIT), sustainable production, small and medium-sized enterprise (SME), adaptation, structural equation modeling (SEM)

## Abstract

Organizational sustainability in the form of environmental management and sustainable production is becoming more important for small and medium-sized enterprises (SMEs) throughout the world. This research evaluates the factors affecting the understanding of the CEO's and managers' intention to adopt practices of green supply chain management (GSCM) and clean innovation technology (CIT) in the manufacturing SMEs of Pakistan. This empirical research identifies key determinants influencing the adoption of GSCM practices. Using structural equation modeling (SEM), we selected a sample size of 350 different manufacturing firms in Pakistan. The results of the study revealed that six factors, namely, environmental, government, organization, suppliers, market, and operational factors, significantly influence the intention to adopt GSCM and positively impact sustainable production. The study's findings reveal that market and operational factors are highly significant for adopting GSCM practices at a *p*-value of 0.05. Environmental and organizational factors are equally significant to adopt GSCM practices at a *p*-value of 0.10. This research also analyzed CIT as a moderator between environmental, government, organization, customer, supplier, economic, market, and operational factors in the context of Pakistan. Hypotheses H9a, H9b, H9f, and H9g were validated and support the use of CIT to boost enterprise production and consumption. The research findings would help policymakers understand how to implement GSCM practices and guide enterprises to implement GSCM and CIT practices for enhancing enterprise performance and environmental sustainability.

## Introduction

Green supply chain management (GSCM) practices are important for firms because they help in increasing profitability by reducing environmental and health risks, which are major customer concerns, and make a firm more sustainable and resilient (Shekarian et al., 2022). The firm can reduce the long-term cost and risk of its supply chain which can help to minimize its impact on the environment and public health. In addition, through the adoption of GSCM, organizations can shape the future of how businesses will be managed, reducing costs, and boosting sustainable products (Darwish et al., 2021). Green supply chain management is a relatively new concept. But in the present scenario, the health of the environment and its sustainability is a major concern for all enterprises. Businesses face two major challenges that are responsible for the lack of people's interest in GCSM. First, is the standardization of the practices, and second, is the lack of a self-regulatory environment (Rupa and Saif, 2022).

Worldwide, economic growth, sustainable production, environmental degradation, as well as high-quality output have been a priority for many countries (Liu et al., 2018). Business enterprises of all sizes try to produce environment-friendly products using environment-friendly methods. The enterprise's performance in environmental management and sustainable production and consumption is essential for small and medium-sized enterprises (SMEs) (Younis and Sundarakani, 2020; Lu et al., 2022; Selviaridis and Spring, 2022). Currently, increasing production and consumption have led to a deterioration of the environment and a variety of health problems, pollution, water waste, and excess carbon emissions. In this context, CEOs and other top managers are being driven to reevaluate their supply chain strategy for sustainable production and consumption.

The adoption of GSCM practices can benefit from the latest scientific and technological achievements in sustainable production and consumption in the organization. In this context, organizations should ask the following questions to influence the intention of CEOs and senior managers to adopt the GSCM practices: Has the adoption of GSCM practices had an impact on sustainable production and consumption? If so, what are the factors? What is the impact of adopting innovative technologies on income and business profit? This research aims to provide scientific answers to these questions to not only clarify scientifically how the adoption of GSCM practices increases organizations' production but also suggest policy measures that will help adopt GSCM practices in SMEs in Pakistan. Due to rapidly increasing globalization, GSCM and clean innovation technology (CIT) have greater importance and are environmentally friendly for manufacturing SMEs (Tachizawa and Wong, 2015; Nayak, 2020; Zhang et al., 2021). The adoption of GSCM and CIT practices has a substantial impact on the development of enterprises with benefits as well as lower energy costs. It also plays a key role in the development of sustainable production, green innovation, environmental sustainability, and the improvement of relationships between suppliers and customers (El Baz and Iddik, 2022; Liu et al., 2022). The adoption of GSCM and CIT encourages sustainable production and consumption for manufacturing SMEs. Despite the increasing popularity of GSCM and CIT in many countries, some issues still need to be addressed. In addition, most studies have focused on large enterprises adopting the GSCM, but in our study, manufacturing SMEs were explored for adopting GSCM and CIT.

The adoption of GSCM and CIT has great importance for profit-generating activities, business developments, environmental impact, and the production of enterprises (Zhang and Zhao, 2022). In many enterprises, the practices of supply chain management and environmental management are intertwined *via* GSCM as long-term operations will increase the competitiveness of the enterprises. Since the use of the GSCM and CIT provides more advantages and influences friendly relationships with both suppliers and consumers internally and externally, the implementation of GSCM and CIT has a significant impact on enterprises' performances and product development. Green supply chain management contributes to corporate CIT and sustainable production and increases the enterprise's efficiency (Li et al., 2020). The adoption of GSCM and CIT boosts enterprise development, reduces environmental issues, and increases the level of production and consumption. Multiple studies have indicated a positive relationship between GSCM and CIT implementation (Zaid et al., 2018). In the manufacturing industry, many factors like awareness of consumers, quality of resource management, stakeholder pressure, and government support influence the adoption of GSCM and CIT for sustainable production and consumption (Jum'a et al., 2021; Banik et al., 2022).

The adoption of GSCM and CIT has been studied in many countries, but there is still a gap in the literature about its adoption in developing countries like Pakistan. In Pakistan, it is generally ignored, especially its impact, and research on the relationship between GSCM and CIT is scarce. The existing literature focuses on specific dimensions such as supplier cooperation, green consumption, and environmental management in organizations. To the best of our knowledge, the adoption of GSCM and CIT for manufacturing SMEs in Pakistani is a new research topic, and this is the first study of its kind to examine the factors influencing the decisions on GCSM. The main purpose of this study is to identify critical success factors for manufacturing SMEs in Pakistan to adopt GSCM and CIT. The results of this study will fill the gap to identify critical success factors that affect the intention of CEOs to adopt GSCM and CIT in their companies. Finally, this study also helps decision-makers to understand the adoption of the GSCM and support SMEs to implement CIT.

## Literature review

### Green supply chain management

The adoption of GSCM is significant in many sectors of the enterprise including human resources, management support, environmental friendliness, business enhancement, sustainable production, customer satisfaction, perceived relative advantage, and cost reduction (Luthra et al., 2015; Zaid et al., 2018; Zhang and Zhao, 2022). It is beneficial for sustainable production by encouraging the market to become more environment-friendly, attracting new workers to the company, and helping the enterprise to establish new business opportunities in both developed and developing countries (Li et al., 2020). Pourjavad and Shahin (2020) investigated the risk of green supply chain management in the pipe industry of Iran for the fuzzy environment. They concluded that the green supply chain was very important for the improvement of the environment within the company. Their findings indicated that GSCM was beneficial for saving resources, increasing environmental and operational capabilities, and helping to reduce costs and environmental degradation. Banik et al. (2022) identified critical success factors for the implementation of GSCM in the electronics industry. The results of the study demonstrated that the adoption of GSCM had a significant impact on sustainable production and consumption, enterprise efficiency, and environmental improvement in developed and developing countries.

Al Khattab and As'ad (2015) deliberated on the relationship between GSCM and environment-based firm performance. Their findings revealed that GSCM had a significant impact on the environment-based firm performance and increased the overall production and consumption of the firm. Yavari and Ajalli (2021) explored the green-resilient supply chain network under the risk of disruptions. According to the study by Sharma et al. (2017), firms were embracing GSCM practices to improve their brand image and increase their market share, and production. Their findings indicated that GSCM practices increased the production of the firms and improved their business.

Many industries adopt GSCM practices to enhance production and improve environmental performance. Similarly, Al-Ghwayeen and Abdallah (2018) explained the adoption of GSCM practices across manufacturers. Kalyar et al. (2020) identified three trends among industrial businesses opting for GCSM: early adopters, followers, and laggards. According to their study, early adopters gained substantial and favorable benefits in environmental, operational, and economic performance. Mondal and Giri (2022) explored the green closed-loop supply under the government subsidy for substitutable products and found that the government subsidy was beneficial for the development of the company.

The adoption of GSCM practices, therefore, is beneficial for sustainable production and encourages the market to become more environment-friendly, attracts new workers to the company, and helps the organization establish new business opportunities in developed and developing countries. A review of the literature shows that the adoption of GSCM and CIT had a significant impact on many aspects of the organization such as human resources, management support, environmental friendliness, business enhancement, sustainable production, customer satisfaction, perceived relative advantage, and cost reduction. Hence, the adoption of GSCM practices and CIT has become a significant topic and of interest to academics in the context of SMEs in Pakistan, along with sustainable production, consumption, and environmental improvement.

### Research gap and highlights

Table 1 provides a comparative overview of the contributions of this paper and previous research. This is for the first time that a analyzes eight factors that affect the adoption of the GSCM practices utilizing CIT as a mediator in the context of manufacturing SMEs in Pakistan. Currently, no study in Pakistan has utilized CIT as a mediator. In addition, research exploring the relationship between the adoption of GSCM and CIT is scarce in Pakistan. The existing literature focuses on specific dimensions such as supplier cooperation, green consumption, environmental management in organizations, and other factors. The adoption of GSCM and CIT in manufacturing SMEs in Pakistan is a new research topic to the best of our knowledge and this is the first study of its kind to examine the factors affecting this decision. The results of this study will fill the research gap and identify critical success factors that can influence the decisions of CEOs to adopt GSCM and CIT in their companies. It will also help decision-makers understand the benefits of adopting GSCM and support SMEs to implement CIT.

**Table 1:** The characteristics of this paper.

**Literature Country GSCM CIT**				**Factor affecting the adoption of GSCM**							
				**Env.F**	**Gov.F**	**Org.F**	**Cust.F**	**Sup.F**	**Eco.F**	**Mkt.F**	**Op.F**
Tian et al. (2014)	China	✓		✓							
Mitra and Datta (2014),	India	✓		✓							
Luthra et al. (2015)	India	✓			✓						
Lee et al. (2018)	Korea	✓	✓								
Mumtaz et al. (2018)	Pakistan	✓		✓							
Wang and Feng (2019)	China		✓						✓		
Zhang et al. (2021)	China	✓							✓		
Deng et al. (2022)	China	✓	✓								
Li et al. (2020)	China	✓		✓							
Liu et al. (2021)	China	✓		✓							✓
Zastempowski and Cyfert (2021)	Poland	✓	✓	✓							
This Paper	Pakistan	✓	✓	✓	✓	✓	✓	✓	✓	✓	✓

### Factors affecting the intention to adopt GSCM and hypotheses developments

#### Environmental factor

Environmental factors play an important role in the production, consumption, and development of enterprises that are committed to sustainability. Enterprises are more aware of implementing environment-friendly practices and processes to reduce their effect on the environment and increase sustainable production (Khan et al., 2019). The term “environment” refers to an enterprise's overall responsibility for its long-term viability. Environment-friendly efforts across an enterprise's GSCM may help the enterprise to enhance its environmental performance and green innovation (Kalyar et al., 2020; Wang et al., 2021). The adoption of GSCM practices minimizes environmental impact, increases production, and helps balance environmental and economic sustainability (Tian et al., 2014). Sustainable production and consumption, as well as waste management, are all considered to be key drivers for enterprises seeking to enhance their environmental performance and efficiency within the context of the GSCM framework (Laari et al., 2017). The adoption of the environmental factor must be included in strategic planning from top to bottom to achieve success and sustainable production. The long-term survival of the enterprise depends on the support of the top management in adopting and implementing new innovative technologies, programs, and activities. Environmental excellence begins with the design of products and processes. The CEO's and management's commitment to an environmentally sustainable strategy is necessary to ensure that GSCM is adopted. The environmental performance advocated in this research includes efforts such as increasing the enterprises' profit, minimizing waste, pollution, and greenhouse gas emissions and adopting GSCM for sustainable production and consumption. This study uses the same indicators of environmental performance as other studies, including environmental factors that support the enterprise's productivity and intention to adopt GSCM, which ultimately improves environmental performance. Therefore, the following hypothesis is proposed:

***H1:*** *Environmental factors directly and positively affect the intention to adopt GSCM*.

#### Governmental factor

Across the world, governments have implemented a range of policies and initiatives to improve enterprises' performance, living standards, and economic development (Tachizawa and Wong, 2015). The government not only subsidizes enterprises' purchases of renewable energy equipment but also provides sustainable energy for the development of enterprises. Kuo et al. (2016) examined the impacts of various carbon tax policies on enterprises' investments in new innovative technology. Meng et al. (2021) found that government subsidies could reduce green products' prices and benefit the manufacturer. Sun and Li (2021) established that governments' participation could accelerate the green behavior of logistics enterprises. The conclusions from these studies demonstrate that government subsidies and new environment-friendly policies are important factors in promoting enterprises' performance and their adopting GSCM practices. The following hypothesis is proposed because of the foregoing assumptions:

***H2:*** *Governmental factors directly and positively affect enterprises' intention to adopt GSCM*.

#### Organizational factor

An organization's internal factors determine the features that are used to evaluate and compare the performance of the enterprise. Human resource management, technology, organizational goals, and vision statements are all examples of internal company factors (Zhang et al., 2020). Production capacity, marketing strategy, management, the age of the enterprise, the expertise of the owner/manager, and organizational culture, all have a major influence on the internal organization of a company (Al-Swidi et al., 2021). The strength among employees is a critical internal business component since it is directly tied to the success of an organization. Employees who are devoted, passionate, and welltrained make a significant contribution to the success of the enterprise. The internal factors of the organization contribute to adopting GSCM. Taking cognizance of the influence of an organization's internal dynamics, the following hypothesis is proposed:

***H3:*** *Organization factors directly and positively affect the intention to adopt GSCM*.

#### Customer factor

The customer is also an important factor in the enterprise's development and performance (Feng et al., 2018). Customer satisfaction has emerged as a critical component in business strategy because higher levels of customer satisfaction are associated with increased customer loyalty, which may result in increased profits for the enterprise (Bowen and Chen, 2001). Lv and Li (2021) studied the influence of green consumers on enterprises' green innovation. Customers, therefore, play an essential part in GSCM. Previous research has established that customer cooperation has a positive impact on production and consumption as well as the development of the enterprise. The customers can help improve and promote businesses by influencing the company's intention to adopt GSCM and CIT. In turn, the adoption of GSCM and CIT can also increase customer satisfaction levels. Considering these statements, the following hypothesis is proposed:

***H4:*** *Customer-related factors directly and positively affect the intention to adopt GSCM*.

#### Suppliers factor

The suppliers provide manufacturing enterprises with raw materials, components, services, and commodities, either directly or indirectly. The suppliers are regularly involved industrial enterprises in the environmental effects through their raw materials as well as in improving the enterprises' production. The supplier factor is very important to adopt GSCM practices. Mumtaz et al. (2018) investigated that the supplier had a significant relationship with the adoption of GSCM practices. The participation of the supplier in GSCM implementation practices is positively significant since the supplier is responsible for environmental standards in material management and processes, as well as buying strategies (Lee et al., 2018). Green supplier collaboration influences enterprises' performance under specific conditions (Feng et al., 2020). Selecting the correct supplier can significantly help companies to be more socially innovative and obtain sustainable production targets (Alavi et al., 2021; Petrudi et al., 2021). The collaboration between an enterprise and its suppliers to accomplish environmental goals and adopt GSCM will facilitate better implementation of GSCM practices for sustainable production. Therefore, the following hypothesis is proposed:

***H5:*** *Supplier-related factors directly and positively affect the intention to adopt GSCM*.

#### Economic factor

The economic factor is very important for the development of enterprises. Economic performance has been established by economists to minimize the cost of enterprises as well as increase profit and production. The economic factor helps to increase an enterprise's profit, reduce its extra costs, and influence the mitigation of the industrial climate in some countries (Wang and Feng, 2019). Consumer behavior, job concerns, interest rates, banking, and inflation are all common economic aspects that influence business development. According to the research by Zhu et al. (2017), customers and suppliers can both improve their environment-friendly performance as well as their economic performance through the adoption of GSCM.

The adoption of GSCM helps to minimize the environmental cost as well as the enterprise's production cost. It has a positive relationship with the enterprise's performance (Bag et al., 2021). The enterprise using GSCM has more profit and benefits compared to enterprises that are unaware of GSCM practices. The economic performance of enterprises significantly increases the production and overall efficiency of the enterprises. So, the following hypothesis is proposed:

***H6:*** *Economic-related factors directly and positively affect the intention to adopt GSCM*.

#### Marketing factor

In many enterprises, a marketing strategy is an important tool for the overall performance of the enterprise. The marketing factors, including pricing, distribution, promotion, and adaptation, have a significant effect on the customers and sustainable production and consumption. “Marketing performance measurement” is the evaluation of the relationship between marketing activities and an enterprise's performance. Abdeljawed and Amraoui (2021) studied the low-voltage DC microgrid technology for energy trading markets. Valor et al. (2022) proposed a strategy to expand the clothing market. In these studies, the marketing strategy had a significant relationship with GSCM. The adoption of GSCM and the marketing factor had a significant relationship to enhance the enterprise's performance, as well as sustainable production and consumption. Therefore, the following hypothesis is proposed:

***H7:*** *Marketing factors directly and positively affect the intention to adopt GSCM*.

#### Operational factor

The operational factor is also very important in the enterprise's performance. An enterprise's operations can be defined as the “management of systems and procedures involved in the manufacture of goods” (Chen et al., 2012). Many operational factors control businesses and increase the production of the enterprise. Operational factors in the enterprise are the key to the enterprise's development, which affects the strategies of decision-makers (Shen and Yu, 2009). The operational factor improves environmental and economic performances. They have a significant relationship with GSCM and the enterprise's production and consumption (Feng et al., 2018). Green supply chain management practices also help to increase the performance of the operational factors (Li et al., 2020; Lim et al., 2022). So, the following hypothesis is proposed:

***H8:*** *Operational factors directly and positively affect the intention to adopt GSCM*.

### Clean innovative technology (CIT)

Clean innovative technology is one of the most essential drivers of long-term success in the enterprise's operation, particularly in dynamic markets and the development of the enterprise. It improves the enterprise's performance and produces new products to enhance sustainable production and consumption (Seman et al., 2019). According to several studies, CIT has been related to sustainable production and consumption, as well as enterprise performance (Zhou et al., 2021; Zhang et al., 2022). It benefits from GSCM and influences SMEs' ability to react quickly to changes in the environment (Zastempowski and Cyfert, 2021; Deng et al., 2022). Furthermore, CIT has become an important development direction to promote sustainable production, consumption, and enterprise efficiency. It represents the technological advancement and intention to adopt GSCM practices for sustainable production and consumption and focuses on innovative ideas and marketing new items that promote environmental sustainability. Given the pivotal importance of CIT in the various factors that influence the decision to adopt GSCM, the following hypotheses are proposed:

***H9a:*** *CIT directly and positively moderates the relationship between environmental issues and CEOs' intention to adopt GSCM*.
***H9b:*** *CIT directly and positively moderates the relationship between governmental issues and CEOs' intention to adopt GSCM*.
***H9c:*** *CIT directly and positively moderates the relationship between organizational issues and CEOs' intention to adopt GSCM*.
***H9d:*** *CIT directly and positively moderates the relationship between customers' issues and CEOs' intention to adopt GSCM*.
***H9e:*** *CIT directly and positively moderates the relationship between suppliers' issues and CEOs' intention to adopt GSCM*.
***H9f:*** *CIT directly and positively moderates the relationship between economic issues and CEOs' intention to adopt the GSCM*.
***H9g:*** *CIT directly and positively moderates the relationship between marketing issues and CEOs' intention to adopt GSCM*.
***H9h:*** *CIT directly and positively moderates the relationship between operational issues and CEOs' intention to adopt GSCM*.

## Research methodology of the study

The manufacturing SMEs are essential components of Pakistan's economy and they have the potential to contribute to more than 35% of the value-added share in the economy. In Pakistan, about 90% of SMEs are privately owned and are recognized as the backbone of the state economy, with a share of almost 40% of the country's GDP, 30% of exports, and 80% of total employment. The sampling frame includes data from CEOs of manufacturing SMEs located in Pakistan's industrial zone. To collect data for this research, the survey used a well-administered questionnaire approach and a face-to-face interview. This research was conducted in 2021 with support from China's National Science Foundation NSF, and a sample size of 350.

The study used measuring items connected to latent variables that had previously been shown to be reliable and valid in earlier studies. The characteristics of the respondent are seen in Table 2 and included the respondent's age, firm experiences, education level, and current position of the respondents in the organization.

**Table 2:** Characteristics of the respondents.

**Variables**	**Sub–attributes**	**Frequency (*n*)**	**Percentage (%)**
Sex	Male	350	99.99
Age	25–35 years	15	4.2
	36–45 years	35	10
	46–55 years	200	57
	Above 55 years	100	28
Firm Experience	Less than 5 years	45	12
	5–10 years	205	58
	More than 10 years	100	28
Education	Bachelor's degree	150	42
	Master's degree and above	200	57
Current Position	CEO	350	99.99

The survey data of the manufacturing SMEs in Pakistan (2021).

Table 3 details the construct and measurement elements. The measurement items related to environmental, governmental, organization, customer, supplier, economic, market, and operational factors are listed. In this study, all measurement items were measured using a 5-point Likert scale (strongly disagree “1” to strongly agree “5”) (Mitra and Datta, 2014).

**Table 3:** Construct and measurements item.

**Construct**	**Measurements Item**	**Scale**	**Source**
Environment	E11: We would like sustainable use of natural resources. E12: We would like supportable use of firms equipment's and materials. E13: We would like reusing and recycling construction materials and equipment. E14: We would like properly waste reduction. E15: We would like carbon emissions reduction.	(“5”-point Likert scale from “strongly disagree (“1”) to strongly agree (“5”)	(Khan et al., 2019; Nguyen et al., 2020)
Governments	G11: We would like Gov. financial support to adopt the GSCM. G12: We would like Gov. technical support to adopt the GSCM. G13: We would like Gov. innovative training opportunities to adopt GSCM. G14: We would like anticipation of Gov. rules and regulations. G15: W would like to anticipation of Gov. legislation. G16: we would like to anticipation of Gov. new innovative policies. G17: we would like to anticipate Gov. environmental certifications.		
Organization	O11: We would like to increased employee for adoption of the GSCM. O12: We would like to increased labor productivity to adopt the GSCM. O13: We would like to support from top managers/CEOs to adopt GSCM.		
Customers	C11: We would like to improve environmental performance. C12: We would like customer/client awareness and pressure. C13: We would like to improve the image of the construction industry.		
Suppliers	S11: We would like firms environmental association with suppliers. S12: We would like to provide firm's awareness and friendly environment. S13: we would like firms environmental collaboration with suppliers. S14: We would like collaboration among product designer and supplier.		
Economic	E11: We would like to use additional labor for adoption of GSCM. E12: We would like to additional capital for adoption of GSCM. E13: We would like additional setup cost for adoption of GSCM. E14: We would like entrepreneurship for adoption of GSCM. E15: We would like additional staff for adoption of GSCM.		
Market	M11: We would like to increase the values of property. M12: We would like to provide tax incentives. M13: We would like to establish firms green image. M14: We would like to decreased infrastructure strain.		
Operational	OP11: We would like increase the product services and design. OP12: We would like to decreased the cost of operation. Op13: We would like to provide new equipment's and technology.		
Intention to adopt GSCM	INT11: We would like that our firm intends to adopt GSCM. INT12: We would like that in future our firm adopt the practices of GSCM. INT13: We would like thatour firm highly recommend to adopt GSCM for other companies.		(Liu et al., 2020)

Construct and measurement items of the research study.

**Figure 1:**
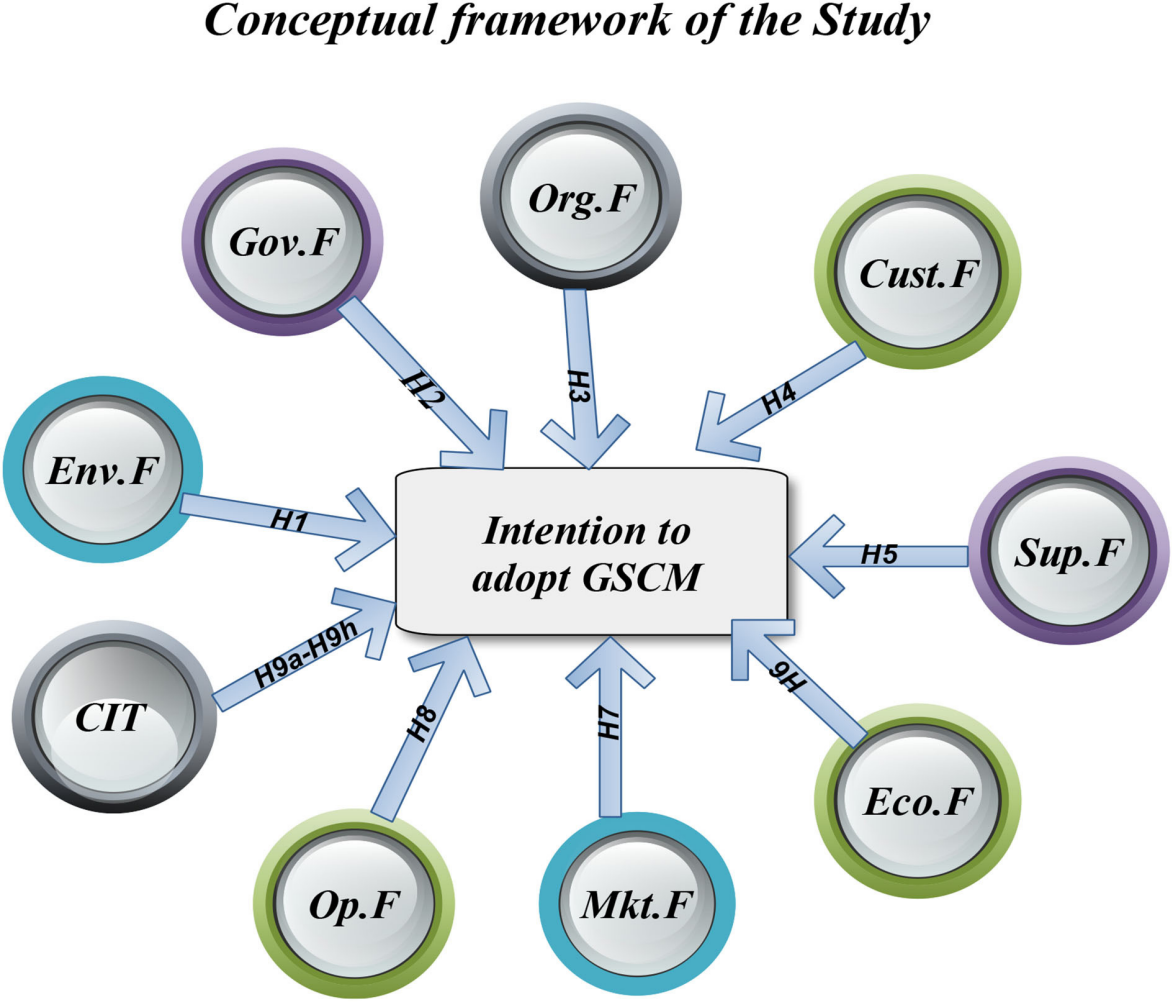
The conceptual framework of the study's hypotheses development. Env.F, Environmental Factor (H1) → Intention to adopt GSCM; Gov.F, Governmental Factor (H2) → Intention to adopt GSCM; Org.F, Organization Factor (H3) → Intention to adopt GSCM; Cust.F, Customer Factor (H4) → Intention to adopt GSCM; Sup.F, Supplier Factor (H5) → Intention to adopt GSCM; Eco.F, Economic Factor (H6) → Intention to adopt GSCM; Mkt.F, Market Factor (H7) → Intention to adopt GSCM; Op.F, Operational Factor (H8) → Intention to adopt GSCM; CIT, CIT (H9a–H9h) → Intention to adopt GSCM.

The data were first examined in SPSS version 25 using descriptive statistics and a multicollinearity analysis. Following that, structural equation modeling (SEM) was utilized to assess the measurement items and structural models. The outcomes of the structural model analysis were used to verify and test the hypotheses. The SEM is a flexible approach to estimating observed variables that depend on latent variables. It represents a significant advancement in regression and path analysis techniques in that it allows estimating the relations between latent variables and observed variables and their relations to other latent variables (Mitchell, 1992). The SEM method is a multivariate technique for testing and assessing multivariate causal linkages that are increasingly being utilized in scientific research. The conceptual framework of the study (Figure 1) provides an overview of the interaction between all the important factors and the CIT to adopt GSCM practices.

## Results and discussions

Table 4 provides the descriptive statistics of the study which includes the total number of observations (N), the value of the latent variable, mean statistics, standard deviation, as well as the value of “skewness and kurtosis. In this research, the value of skewness and kurtosis are used for the normalcy of the given data.

**Table 4:** Descriptive statistics of the study.

**Variables**	**N Statistics**	**Means Statistics**	**S.D Statistics**	**Skewness**		**Kurtosis**	
				**Statistics**	**Std. error**	**Statistics**	**Std. error**
Environment Factors	350	3.95	0.88	−1.431	0.130	2.103	0.260
Government Factors	350	3.27	1.13	−-0.389	0.130	−1.006	0.260
Organization Factor	350	3.03	1.17	−0.092	0.130	−1.157	0.260
Customers Factors	350	3.67	1.29	−0.779	0.130	−0.811	0.260
Suppliers Factors	350	3.74	1.12	−1.036	0.130	−0.024	0.260
Economically Factors	350	3.56	1.27	−0.814	0.130	−0.810	0.260
Market Factors	350	3.12	1.18	−0.432	0.130	−1.026	0.260
Operational Factors	350	3.29	1.24	−0.508	0.130	−1.094	0.260
Intentiontoadopt GSCM	350	3.38	1.18	−0.532	0.130	−0.787	0.260

Authors' own calculation from the survey data of SMEs in Pakistan (2021).

**Figure 2:**
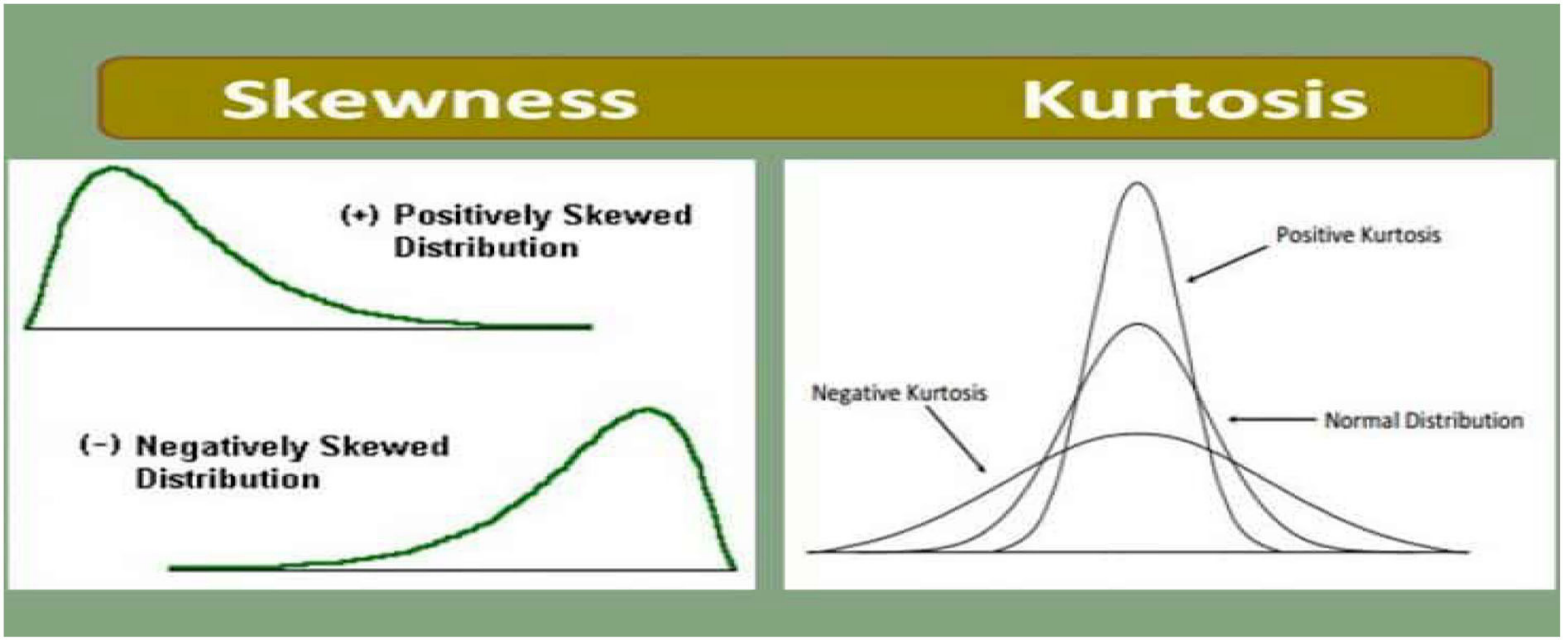
The skewness and kurtosis distributions.

If the skewness value tilts to the left, it means a negative value. On the other hand, a tilt to the right indicates positive skewness. Kurtosis is a term used to describe data that has either peaksoris flat (Khorasani et al., 2014). As seen in Figure 2, the negative kurtosis value indicates the distribution is flatter than a normal curve with the same mean as well as standard deviation. Similarly, a positive kurtosis value indicates that the distribution is peaked and has a long tail. The values ranging from −3 and +3 show the skewness fall while the kurtosis ranges from −10 to +10. The results presented in Table 4 indicate that the environmental factor has the greatest mean value (Mean = 3.95), while the organizational factor has the lowest mean value (Mean = 3.03).

Table 5 lists the multicollinearity statistics and presents how the many independent variables correlate with each other in the model. The statistical analysis' path coefficients may change due to the significant correlations between the independent variables. In this study, the variance inflation factor (VIF) and tolerance levels were used to analyze and identify multicollinearity (Senaviratna and Cooray, 2019). The findings of the study indicate that the VIF values of environmental factors, government factors, organizational factors, customer factors, economic factors, market factors, and operational factors are less than 5 and tolerance values of all the factors are more than 0.10 as shown in Table 5. Hence, the results demonstrate that there is no multicollinearity in this study.

**Table 5:** Collinearity statistics.

**Variables**	**Intention to adopt GSCM**	
	**Tolerance**	**VIF**
Environment factors	0.788	1.269
Government factors	0.456	2.192
Organization factors	0.744	1.343
Customers factors	0.565	1.771
Suppliers factors	0.809	1.236
Economic factors	0.942	1.062
Market factors	0.650	1.538
Operational factors	0.882	1.134

Authors' own calculation from the survey data of SMEs in Pakistan (2021).

The results of the convergent validity and reliability are shown in Table 6. In this study, we utilized Cronbach's alpha (CR) and composite reliability to analyze and test the latent variables. The significant value of CR is more than 0.7 which indicated the latent variables are very dependable.

**Table 6:** Results of estimating the measurement model.

**Variables**	**Items**	**Factor loadings**	**Cronbach' salpha**	**Composite reliability**	**Average variance extracted (AVE)**
Environmental Factors	E11	0.67	0.85	0.85	0.54
	E12	0.72			
	E13	0.84			
	E14	0.75			
	E15	0.71			
Government Factors	G11	0.75	0.91	0.90	0.59
	G12	0.78			
	G13	0.73			
	G14	0.77			
	G15	0.74			
	G16	0.86			
	G17	0.74			
Organization Factors	O11	0.66	0.80	0.80	0.58
	O12	0.83			
	O13	0.79			
Customers Factors	C11	0.95	0.89	0.89	0.74
	C12	0.80			
	C13	0.84			
Suppliers Factors	S11	0.69	0.88	0.88	0.66
	S12	0.87			
	S13	0.85			
	S14	0.83			
Economics Factor	E11	0.88	0.93	0.93	0.73
	E12	0.84			
	E13	0.87			
	E14	0.84			
	E15	0.85			
Market Factors	M11	0.64	0.87	0.84	0.59
	M12	0.67			
	M13	0.75			
	M14	0.97			
Operational Factors	OP11	0.66	0.82	0.82	0.61
	OP12	0.74			
	OP13	0.93			
Intention to adopt GSCM	INT11	0.59	0.84	0.79	0.57
	INT12	0.89			
	INT13	0.77			

Authors' own calculation from the survey data of SMEs in Pakistan (2021).

In this research, the results show that the CR values are greater than 0.70 in all latent variables, indicating that reliability has been established. The findings also show that convergent validity is determined by factor loading values greater than 0.70 as well as the average variance extracted (AVE), values, which were also greater than 0.50 in this study, suggesting that the research is appropriate.

Table 7 presents the results of the discriminant validity test. From Table 7, we find that the discriminant validity is achieved when the value of the square root (AVE) is greater and higher than the coefficients of correlation among all including constructs. The square root values of the environmental factor, governmental factor, organization factor, customer factor, supplier factor, economics factor, market factor, and operational factor are greater. These results indicate that all latent variables contain higher values as compared to the square root values. Hence the discriminating validity of the latent variables is established.

**Table 7:** Results of discriminant validity.

**Latent variables**	**1**	**2**	**3**	**4**	**5**	**6**	**7**	**8**
Environmental Factor	0.978							
Government Factors	0.288	0.978						
Organization Factors	0.344	0.190	0.929					
Customers Factors	0.390	0.270	0.564	0.921				
Suppliers Factors	0.250	0.488	0.277	0.271	0.949			
Economics Factor	0.333	0.140	0.567	0.532	0.240	0.906		
Market Factors	0.345	0.226	0.562	0.469	0.251	0.520	0.952	
Operational Factors	0.298	0.476	0.081	0.169	0.345	0.047	0.216	0.982

Authors' own calculation from the survey data of SMEs in Pakistan.

The results of the structural model analysis are presented in Table 8, including the *p*-values, *t*-statistics, and the path coefficients of the variables. According to the findings of the study, six out of eight factors have a substantial and favorable influence on adopting GSCM practices. The study's findings reveal that market and operational factors are highly significant to adopt GSCM practices at a *p*-value of 0.05. Environmental and organizational factors are also strongly significant for adopting GSCM practices with a *p*-value of 0.10. The overall finding of this study indicates that hypotheses H1, H2, H3, H5, H7, and H8 are positively significant for adopting GSCM practices for sustainable production and consumption as well as enterprise efficiency.

**Table 8:** Results of estimating the analysis of the structural model.

**Paths**	**Path Coefficient**	**T statistic**	***P*** **-value**	**Results**
Environmental Factors → intention to adopt GSCM (H1)	0.21	3.60	0.001	Significant
Government Factors → intention to adopt GSCM (H2)	0.14	2.41	0.05	Significant
Organization Factors → intention to adopt GSCM (H3)	0.41	4.80	0.001	Significant
Customers Factors → intention to adopt GSCM (H4)	−0.05	−0.93	0.349	Non-Significant
Suppliers Factors → intention to adopt GSCM (H5)	0.31	5.34	0.001	Significant
Economic Factors → intention to adopt GSCM (H6)	0.09	1.87	0.06	Non-Significant
Market Factors → intention to adopt GSCM (H7)	0.26	4.45	0.001	Significant
Operational Factors → intention to adopt GSCM (H8)	0.3	4.97	0.001	Significant
Environmental factors → intention to adopt GSCM (H1)	0.21	3.60	0.001	Significant
Government factors → intention to adopt GSCM (H2)	0.14	2.41	0.05	Significant
Organization factors → intention to adopt GSCM (H3)	0.41	4.80	0.001	Significant
Customers factors → intention to adopt GSCM (H4)	−0.05	−0.93	0.349	Non-Significant
Suppliers factors → intention to adopt GSCM (H5)	0.31	5.34	0.001	Significant
Economic factors → intention to adopt GSCM (H6)	0.09	1.87	0.06	Non-Significant
Market factors → intention to adopt GSCM (H7)	0.26	4.45	0.001	Significant
Operational factors → intention to adopt GSCM (H8)	0.3	4.97	0.001	Significant

Authors' own calculation from the survey data of SMEs in Pakistan (2021).

Table 9 shows the findings of the moderation analysis. Clean innovation technology as a mediator considerably moderates the impact of four variables and strongly influences the use of GSCM practices. It substantially mitigates the influence of governmental and economic variables on the desire to adopt GSCM at a *p*-value of 0.05. The results also indicate that CIT mitigates the influence of market factors on the intention to adopt GSCM at a *p*-value of 0.10. Hypotheses H9a, H9b, h9f, and H9g are all validated and support the use of CIT to boost the enterprise's production and consumption.

**Table 9:** Results of estimating the moderation analysis.

**Paths**	**Path coefficient**	**T statistic**	***P*** **-value**	**Results**
Environmental factors → CIT (H9a)	0.32	0.087	0.632	Significant
Government factors → CIT (H9b)	0.15	2.51	0.05	Significant
Organization factors → CIT (H9c)	−0.35	−0.70	0.421	Non-significant
Customers factors → CIT (H9d)	−0.04	−0.91	0.341	Non-significant
Suppliers factors → CIT (H9e)	−0.70	−1.49	0.123	Non-significant
Economically factors → CIT (H9f)	0.25	2.90	0.05	Significant
Market factors → CIT (H9g)	0.29	4.57	0.001	Significant
Operational factors → CIT	−0.02	−0.047	0.632	Non-significant

Authors' own calculation from the survey data of SMEs in Pakistan (2021).

**Figure 3:**
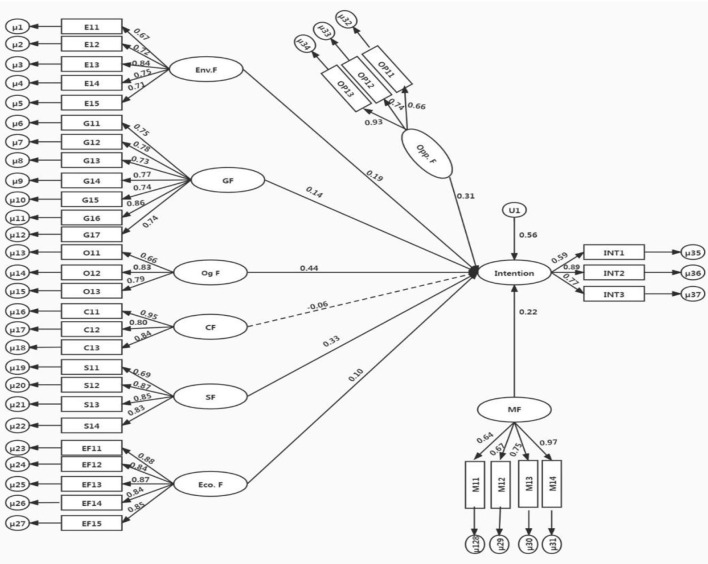
The results of the study.

Figure 3 demonstrates that environmental, governmental, organizational, market, and operational factors significantly influence CEOs' intentions to adopt GSCM practices.

The environmental factor (Figure 3) includes five important items (E11–E15) such as recycling of construction materials, proper waste reduction, carbon emission reduction as well as sustainable construction materials. The highest factor loading value of 0.84 indicates the highest intention to adopt GSCM practices. The governmental factor contains seven items (G11–G17), including initial government financial support, technical support from the government, innovative training from the government, the anticipation of government legislation, rules and regulations, and new innovative policies from the government to adopt GSCM practices. The highest factor loading of the government factor value of 0.86 indicates the highest intention to adopt GSCM practices for sustainable production and consumption. The organizational factor has three items (O11–O13), including increasing employees, increasing labor productivity, and providing support to all workers for CEOs to adopt GSCM practices. The highest factor loading of the organization factor value of 0.83 indicates the highest intention to adopt GSCM practices. The suppliers' factor includes four items (S11–S14) of the firms' environmental association with suppliers, the firm's awareness and friendly environment with suppliers, the firm's environmental collaboration, and product designer collaboration. The highest factor loading of supplier factor value of 0.87 indicates the highest intention to adopt GSCM practices. The market factor includes the four items (M11–M14), containing the ability to increase the values of property, to provide tax incentives, like establishing a firm's green image, and to decrease infrastructure strain by adopting GSCM practices. The operational factor has three items (Op11–Op13) such as increasing the product services and design, decreasing the cost of operation, and the enterprise providing new equipment and technology. It had a factor loading value of 0.93 to adopt GSCM practices. In this research, the moderating effect of CIT was significant in the relationships between CIT and environmental factors, economic factors, governmental factors, and market factors. The overall finding of the study indicated that the adoption of GSCM practices and CIT are beneficial for enterprises' performances.

## Conclusion and policy implications

The purpose of this research was to determine the factors that influence GSCM adoption in Pakistan SMEs and the moderating effect of CIT for sustainable production and consumption. As a result, the research has made significant contributions. For example, the study provides academics and decision-makers with a comprehensive framework that identifies the factors that impact decisions regarding GSCM practices. According to the data, the most significant drivers of GSCM adoption among CEOs are environmental, governmental, organizational, supplier, market, and operational factors. The most important variables are those that are connected to CEOs' intentions to adopt GSCM. The most influential elements are identified in this research, with the organizational component having a major impact, followed by the environmental factor, the governmental factor, the supplier factor, the market factor, and the operational factor.

In this research, the results show that the CR values are greater than 0.70 in all latent variables, indicating that reliability has been established. The findings also show that convergent validity is determined by factor loading values of more than 0.70 as well as average variance extracted (AVE) values greater than 0.50. The AVE was more than 0.50, and all factor loading values were greater than 0.70, suggesting that the research is accurate. We find that the discriminant validity is achieved when the value of the square root (AVE) is greater and higher than the coefficients of correlation among all including constructs. The square root values of the environmental factor, governmental factor, organization factor, customer factor, supplier factor, economics factor, market factor, and operational factor are greater. The results of the study indicate that all latent variables contained higher values as compared to the square root values. Hence the discriminating validity of the latent variables is established.

The overall finding of the study will help the policymakers when they intend to apply GSCM practices and CIT. The findings confirm that CIT positively and significantly moderates the relationship between CIT and other factors such as governmental factors, economic factors, and market factors for sustainable production and consumption. In other words, small manufacturing enterprises are more likely to adopt GSCM practices as compared to large manufacturing enterprises.

The findings from this research also establish that the organizational factor is the most important factor influencing the adoption of GSCM. It can promote the adoption of GSCM and CIT by improving corporate social responsibility and labor productivity. The government plays an equally important role. The government should increase the support for SMEs by providing requisite finance and technology, and formulating relevant policies and regulations to ensure the green transformation of enterprises. In addition, improving social environmental awareness, creating a good business environment and enterprise cooperation, increasing product services and design, reducing operating costs, and establishing an enterprise green brand image can effectively contribute to the green transformation of enterprises.

## Discussions

This study evaluated the critical success factors influencing the adoption of GSCM and CIT in the manufacturing SMEs of Pakistan. The results of the study revealed that six factors, namely, environmental, government, organization, suppliers, market, and operational factors significantly influence the intention to adopt GSCM and positively impact sustainable production. The result of the structural model analysis included the *p*-values, *t*-statistics, and the path coefficients of the variables. It revealed that market and operational factors are highly significant to adopt GSCM practices with a *p*-value of 0.05. Environmental and organizational factors are also strongly significant in influencing decisions regarding GSCM practices with a p*-*value of 0.10. The overall finding of the study indicates that hypotheses H1, H2, H3, H5, H7, and H8 are positively significant to adopt GSCM practices for sustainable production and consumption as well as enterprise efficiency. The study also found that CIT as a mediator considerably moderates the impact of four variables and strongly influences the use of GSCM. Clean innovation technology substantially mitigates the influence of governmental and economic variables on the desire to adopt GSCM with a *p*-value of 0.05. The results also indicate that CIT mitigates the influence of market factors on the intention to adopt GSCM with a *p*-value of 0.10. The adoption of GSCM and CIT has gained contemporary research significance in the context of Pakistan SMEs, and this study would be of interest to academics as well as stakeholders, including policymakers, invested in sustainable production and consumption, and environmental improvement.

### Limitations and future research

There are several limitations to this study. This empirical research addresses only eight factors that may impact decisions to adopt GSCM practices, and future studies should consider other parameters. Also, this research only looked at CIT as a mediator; future studies could consider including other social demographic characteristics as moderators, which might generate new and different results.

## Data availability statement

The original contributions presented in the study are included in the article/supplementary material, further inquiries can be directed to the corresponding author.

## Ethics statement

Ethical review and approval was not required for the study on human participants in accordance with the local legislation and institutional requirements. Written informed consent from the (patients/participants or patients/participants legal guardian/next of kin) was not required to participate in this study in accordance with the national legislation and the institutional requirements.

## Author contributions

SM reviewed the literature, proposed the research model, and designed the study. YZ conducted the literature search, proceeded with the data extraction process, was involved in the development of the manuscript, put forward many constructive suggestions for revising the manuscript, and supervised the entire writing process. SM and IJ conducted the statistical analysis and revised the manuscript critically for important content. YZ, XL, and SH revised the whole manuscript according to the comments of the reviewer and rechecked the relevant data of the manuscript. IJ and KS participated in the writing of the first manuscript. All authors have equal contributions and approve the final manuscript to be published.

## Funding

Financial support was given by the National Science Foundation of China (Grant No. 71901129).

## Conflict of interest

The authors declare that the research was conducted in the absence of any commercial or financial relationships that could be construed as a potential conflict of interest.

## Publisher's note

All claims expressed in this article are solely those of the authors and do not necessarily represent those of their affiliated organizations, or those of the publisher, the editors and the reviewers. Any product that may be evaluated in this article, or claim that may be made by its manufacturer, is not guaranteed or endorsed by the publisher.
